# Dementia Patient Segmentation Using EMR Data Visualization: A Design Study

**DOI:** 10.3390/ijerph16183438

**Published:** 2019-09-16

**Authors:** Hyoji Ha, Jihye Lee, Hyunwoo Han, Sungyun Bae, Sangjoon Son, Changhyung Hong, Hyunjung Shin, Kyungwon Lee

**Affiliations:** 1 Lifemedia Interdisciplinary Program, Ajou University, Suwon 16499, Korea; hjha0508@ajou.ac.kr (H.H.); alice0428@ajou.ac.kr (J.L.); ainatsumi@ajou.ac.kr (H.H.); roah@ajou.ac.kr (S.B.); 2 Department of Psychiatry, Ajou University School of Medicine, Suwon 16499, Korea; sjsonpsy@ajou.ac.kr (S.S.); antiaging@ajou.ac.kr (C.H.); 3 Department of Industrial Engineering, Ajou University, Suwon 16499, Korea; shin@ajou.ac.kr; 4 Department of Digital Media, Ajou University, Suwon 16499, Korea

**Keywords:** digital health, dementia, bioinformatics, multidimensional data visualization, visual analytics, design studies, big data

## Abstract

(1) Background: The Electronic Medical Record system, which is a digital medical record management architecture, is critical for reliable medical research. It facilitates the investigation of disease patterns and efficient treatment via collaboration with data scientists. (2) Methods: In this study, we present multidimensional visual tools for the analysis of multidimensional datasets via a combination of 3-dimensional radial coordinate visualization (3D RadVis) and many-objective optimization (e.g., Parallel Coordinates). Also, we propose a user-driven research design to facilitate visualization. We followed a design process to (1) understand the demands of domain experts, (2) define the problems based on relevant works, (3) design visualization, (4) implement visualization, and (5) enable qualitative evaluation by domain experts. (3) Results: This study provides clinical insight into dementia based on EMR data via visual analysis. Results of a case study based on questionnaires surveying daily living activities indicated that daily behaviors influenced the progression of dementia. (4) Conclusions: This study provides a visual analytical tool to support cluster segmentation. Using this tool, we segmented dementia patients into clusters and interpreted the behavioral patterns of each group. This study contributes to biomedical data interpretation based on a visual approach.

## 1. Introduction

### 1.1. Research Background

The Electronic Medical Record (EMR) system has been utilized as a tool by medical experts to enhance data analysis and enable systematic control of medical records. However, medical records and diagnostic variables comprising EMR vary widely. Thus, a system that can facilitate organization of these data is imperative. The EMR analysis includes multidimensional data, because the data contain several variables.

Multidimensional data analytics play an important role in understanding and analyzing EMR data. Analysis of medical records using multidimensional architectures contributes to a prevention-oriented healthcare system and reduces medical expenses. Thus, data visualization experts have conducted various types of research analyzing multidimensional medical datasets via collaboration with clinicians. Data visualization is a redesigned concept of data analysis with better readability, offering distinct insights that cannot be grasped from a table or graph. Wang et al. [1] visualized various prognoses associated with renal cancer progression by visualizing renal cancer data. In addition, LifeLines and Plaisant et al. [2] proposed a case study by visualizing patients’ medical records and test results to facilitate patients’ medical examination results and treatment schedules.

According to a study of Lhuillier et al. [3], investigators, by administering a language test for patients with Alzheimer’s disease, can detect inaccuracies and incorrect answers systematically by visualizing their test results. 

Likewise, visualization via medical collaboration elucidates complex medical datasets [4]. The field of psychiatry has used cluster analysis as a primary data analytical tool. For example, the factors contributing to eating disorders in young women were identified using Ward’s methods, a cluster analytical tool [5]. Moreover, Petrovic et al. [6] proposed a method of cluster segmentation utilizing neuropsychiatric inventories of patients with dementia. In conclusion, the combined interventions of multidisciplinary experts can enhance the quality of medical data and systems.

### 1.2. Study Goals and Research Process

Using preexisting case studies, this study aims to develop a visual analytical tool to assist psychiatrists in analyzing multidimensional medical datasets.

We followed the following design processes. (1) Understanding and analysis of psychiatrists’ demands in accessing multidimensional medical data. (2) Defining problems based on demands. (3) Designing visualization tools for multidimensional data analysis. (4) Implementing visualization. (5) Conducting qualitative evaluations with domain experts (psychiatrists). Considering that domain experts lack experience analyzing multidimensional medical data, case studies were proposed to elucidate the functions of visualization tools. The study contributions can be summarized as follows.

(a) We designed a 3D RadVis to support the analysis of multidimensional datasets and segmentation of patient clusters. We also presented Parallel Coordinates to present patients’ data.
(b) We verified the 3D RadVis visualization tool via qualitative evaluation and case studies.

**Figure 1:**
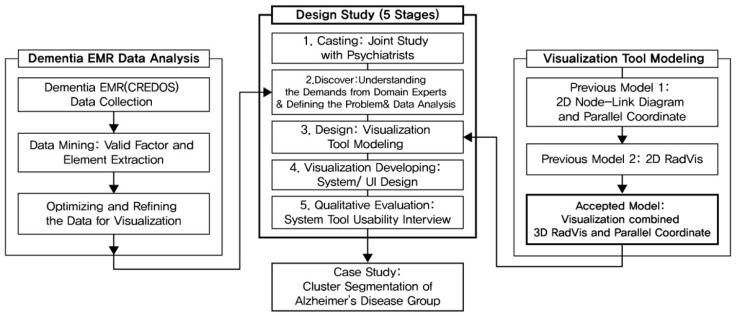
Research framework.

This study was conducted as a user-driven study rather than a technology intervention in other fields of visualization research. The 3D RadVis visualization proposed in this study is based on the studies of Ha et al. [7,8] and Lee et al. [9,10]. Our previous study dealt with the developing process of RadVis and Parallel Coordinates focused on visualization technology. It can alleviate the overlapping nodes shown in 2D RadVis. Figure 1 presents the framework of our study.

## 2. Related Work

In order to understand multidimensional data, it is critical to utilize tools to draw a meaningful conclusion, as individuals generally understand a limited amount of information at once. In the fields of Human–Computer Interaction, Big Data, and Information Visualization, techniques that refine data dimension for visualization have been proposed to enable users understand multidimensional data accurately. Here, we will review published studies of multidimensional data visualization.

### 2.1. RadVis and Parallel Coordinates

RadVis facilitates visualization via location of dataset variables in a circumference loop and presents data samples as nodes inside of the circumference. According to Hoffman et al. [11], this type of visualization leads to arrangement of data nodes from n-dimension dots to a two-dimensional map. In some cases, RadVis visualization is used with a multidimensional data visualization technique. Bertini et al. [12] proposed a method combining 2D RadVis and Parallel Coordinates to visualize multidimensional data. RadVis can be used to analyze the relationship between data clusters and Parallel Coordinates to analyze the numerical values of data. Thus, users not only understand the traits of data clusters, but also simultaneously analyze the variable values of data comprising each cluster. Recently, de Carvalho Pagliosa et al. [13] have suggested a RadViz++ that adds an icicle-plot metaphor to preexisting RadVis visualization, which effectively resulted in a data cluster. However, the aforementioned methods are limited by poor legibility, excessive number of data instances or variables, resulting in overlapping nodes comprising 2D RadVis. Therefore, this study presents a new type of RadVis that prevents overlapping nodes.

Parallel Coordinates has become one of the most widely used methods to visualize multidimensional datasets [14,15]. Assuming that there is *n*-number of variables, each variable is presented as a vertical bar. Data instances are illustrated by a line graph following values contained in each variable in accordance with each vertical bar. Fua et al. [16] have used Parallel Coordinates for visualization based on hierarchical clustering, to represent cluster data. Zhou et al. [17] proposed a clustering method using Parallel Coordinates curves and visual bundles from diverse perspectives to understand cluster patterns. Moreover, using multiple parallel coordinate visualization methods, Tong et al. [18] presented an analytical tool for daily physical activities and sedentary behaviors. However, in these three studies, users can hardly analyze data that are filtered from more than two sections on a single axis.

### 2.2. Cluster Analysis

Cluster analysis is widely used in fields of psychiatry, psychology, social science, and data visualization [19] to segment groups with different traits. For example, Ankerst et al. [20] suggested a multidimensional data visualization tool based on hierarchical clustering. Ismail et al. [21] also conducted a cluster analysis of patients’ traits based on EMR data, developed a system to analyze each cluster, and proposed a relevant algorithm.

In addition, the Stacked Tree of Bission et al. [22] supports visualization of hierarchical clusters containing a maximum of 50,000 nodes. Martínez-Martínez et al. [23] organized cluster classifications based on pie charts and extracted correlations of hierarchical clusters using labeled and colored bars. While the foregoing cluster analysis presents data traits in each cluster, it does not facilitate segmentation or reorganization by users. Therefore, this study designed a function to segment cluster results of data instances that fit users’ analytical goals.

## 3. Research Process

The primary goal of this study was to design a system to help medical experts with data analysis. Thus, we followed a sequential process of “casting (targeting visualization users)–discovery (problem defining)–design (visualization design)–visualization (develop process and visualization interaction)–qualitative evaluation (implementation of visualization system)” to reflect their demands. In addition, during the stages of “Defining demands of experts” and “Qualitative evaluation”, we conducted interviews with medical and clinical experts who studied dementia or were involved in dementia diagnosis. Thus, we elucidated the needs of the analytical tool for medical experts and the efficiency of the actual tool. The methods presented in this study were based on the design methodology suggested by Sedmair et al. [24] and Ghani et al. [25].

The research process comprises five steps: (1) Understanding the demands of domain experts (Section 3.1), (2) defining problems (Section 3.2), (3) designing visualization tool (Section 3.3), (4) develop process of visualization & System Interaction (Section 3.4), and (5) qualitative evaluation by doctors and clinical psychologists (Section 3.5). Following these five steps, the visualization interface was designed to fit the domain experts. This tool supports segmentation of multidimensional diagnostic data of dementia patient group. It delineates the numerical values of variables in dementia behavioral tests.

Moreover, this study devised a 3D type of RadVis layout to prevent nodes from overlapping and adopted a dimensional anchor to define the location of nodes based on an average value of nodes. The dimensional anchor refers to a mid-point in the process where the applied strength of data instance nodes varies in accordance with their values. Dimensional anchors are presented as points at equidistant intervals on a circumference in a 2D RadVis type while they are presented as edges of *z* axis in a cylinder.

### 3.1. Casting

To elucidate the probable demands of cluster segmentation based on dementia patient data, we met two psychiatrists. Furthermore, their major study is mainly focused on topics related to “elderly mental health (anxiety, insomnia, and mental disorder as a result of anger or stress), cognitive impairment, elderly behavior caused by dementia, and senile depression”. Their research career is more than 20 years. To investigate dementia from various perspectives, they have utilized a dementia cohort from Clinical Research Center for Dementia of South Korea (CREDOS) [26]. The changes in dementia patients were analyzed using diverse variables. The findings of the two psychiatrists contribute to the implementation of visualization tool.

### 3.2. Discovery

This study defined the demands of psychiatrists and analyzed previous studies in the fields of medical visualization. We established a design guideline based on the concept of cluster analysis. We analyzed the traits of CREDOS and its advantages.

#### 3.2.1. Understanding the Demands from Domain Experts

Generally, the tests conducted for dementia diagnosis are complex, and vary in cost according to the test type. For these reasons, the medical community has called for a need to develop a simplified model for dementia diagnosis to reduce expenses, based on patient data analysis.

The psychiatrists who participated in this study also hoped to provide treatments adequate to distinguish dementia patient groups according to their symptoms. Further, the characteristics of different dementia patient groups vary widely. These are the difficulties faced by the psychiatrists in evaluating test results and values of dementia patients. In summary, dementia patient analysis based on data visualization can address the foregoing challenges faced by psychiatrists and contribute to improved medical data analysis.

#### 3.2.2. Design Guideline

Based on the demands of the psychiatrists, this study established a system of cluster analysis of dementia patients. To this end, we established a design guideline, as shown in the Table 1 below. The design guideline was utilized to test the visual analysis. In addition, qualitative evaluations of each design task were conducted by psychiatry experts. The system design guideline shown in Table 1 was utilized to create the questionnaire for qualitative evaluation (step 5, Section 3.5) based on the design study.

#### 3.2.3. Analysis System Subject Data: CREDOS

Data used for analysis and visualization tool implementation in this study were based on a dementia-diagnosed patient cohort called CREDOS [26]. Variable information in the CREDOS cohort is presented in Table 2. The cohort data were collected from 37 university hospitals between 2005 and 2013, and involved 21,094 cases of dementia diagnosis. Variables included in the data were mostly tests used to diagnose dementia, such as psychological test, neuropsychological test, neurologic test, drug intake history, and privacy information of patients. The total number of variables was 438. We classified dementia patients using the five steps (SMI, MCI, VCI, SVD, and AD) of diagnosis.

The dementia patients included in the CREDOS were largely segmented into five categories ranging from Subjective Memory Impairment (SMI) to Alzheimer’s Disease (AD) based on the chronic level of dementia. SMI is the most preliminary stage. Mild Cognitive Impairment (MCI) is the second stage representing entry into chronic dementia. The third and fourth stages, including Vascular Cognitive Impairment (VCI) and Subcortical Vascular Dementia (SVD), indicate brain damage and cognitive impairment. Lastly, known as a stereotype of dementia, AD has the widest node distribution in the CREDOS data. Although several diagnostic indices of dementia classification exist in the medical field, this study segmented dementia patient groups based on the five steps of psychological testing for convenience. In addition, we used cognitive test results directly correlated with the cognitive ability of dementia patients as visualization variables.

### 3.3. Design

This study has been subjected to data mining [27,28] to extract the variables that significantly affect the diagnosis of dementia.

We applied the k-Scale variable selection method at the stage of ‘proposer module’, especially in the model of Quad-phased data mining for dementia diagnosis based on the study by Bang et al. [28]. As a result, we extracted 62 primary variables selected from 438 CREDOS data variables for visualization.

To determine the finest strategy to visualize the extracted data, we used various techniques of visualization and based on the results, we determined the pros and cons for each technique. After analysis of the feedback, 3D RadVis and Parallel Coordinates at Section 3.3.3 were finally selected as the model system for visualization.

#### 3.3.1. Previous Model 1: 2D Node-Link Diagram and Parallel Coordinates

**Figure 2:**
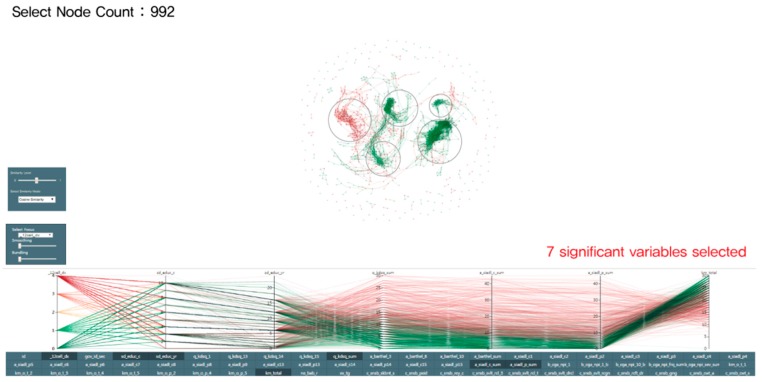
Previous model 1: 2D node-link diagram and Parallel Coordinates.

First, we designed a 2D node-link diagram visualization based on test results in order to elucidate the relationship between data distribution and dementia diagnostic records. Figure 2 illustrates 2D node-link diagram visualization.

Using the node-link diagram, the visualization above highlights the relationship with dementia patients. A single node represents a single patient and patients with similar symptoms are linked. Based on the node-link diagram, the parallel coordinates visualization presents the detailed diagnostic information values of patients [29].

This node-link diagram has been constructed to represent each result based on the similarity between the variables selected in the parallel coordinates. When the user selects the variables, the node-link diagram is calculated by the force-directed graph algorithm. Since this visualization system provides options such as cosine similarity, similar Spearman correlation and Pearson correlation coefficient as well as adjustments (from 0–1), the user can freely balance the values to draw a node-link diagram for the analysis of similarities.

However, poor legibility was detected during the psychiatrist evaluation interviews. Moreover, it was difficult to intuitively understand the traits of each node in the clusters. Nevertheless, we found that it presented the multidimensional dataset of CREDOS and that clusters of dementia patients were variously segmented. We have reported the relevant information as study results [30] and received feedback from data visualization experts. As a result, rather than using the 2D node-link diagram, we concluded that complementing the system with RadVis was more adequate.

#### 3.3.2. Previous Model 2: 2D RadVis

As the secondary step in the visualization method, this study designed a cluster visualization of dementia patients using RadVis technique. In the RadVis visualization, when a specific data variable increased a strong force was applied to the dimensional anchor containing variable information and remove the data instance. However, when a specific variable was decreased, a lesser force was applied to the dimensional anchor [31,32].

**Figure 3:**
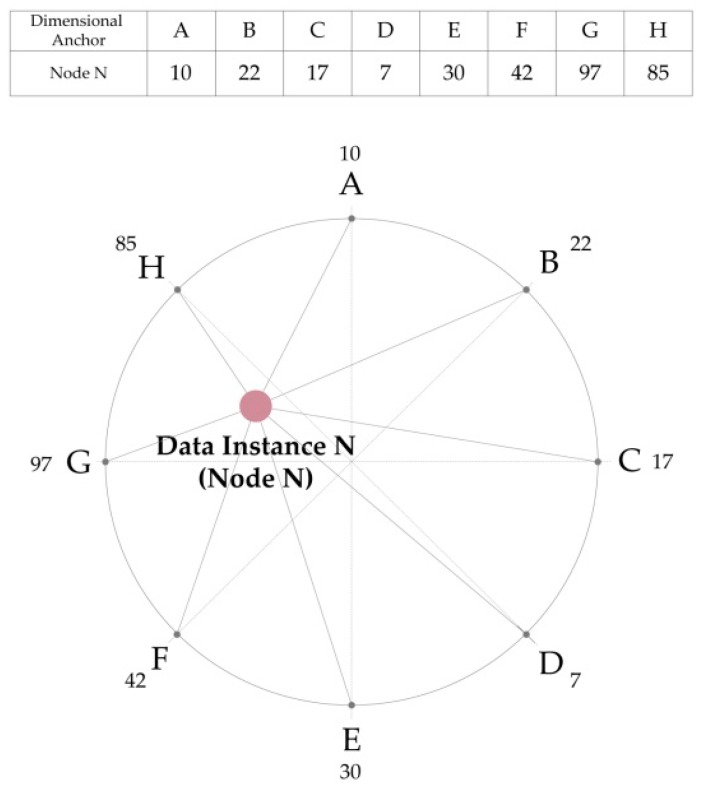
Principle of RadVis Visualization.

Figure 3 illustrates the principle of data instance location in the 2D RadVis. Assuming that a Node N contains data values of eight variables in total (from A–H), each value was the same as shown in the table of Figure 3. Here, we can see that the higher the variable value of the node N, the stronger was the dimensional anchor pulling the node N.

**Figure 4:**
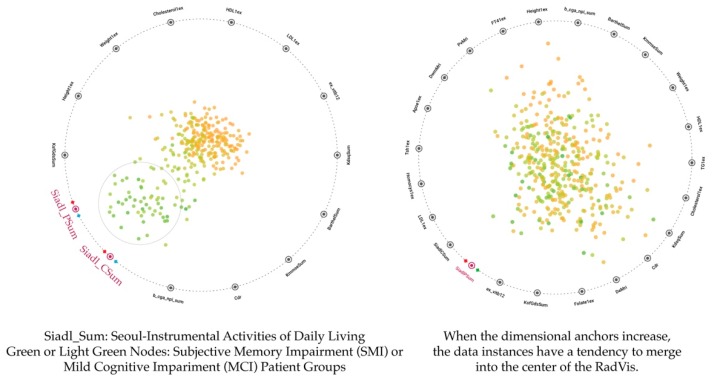
Previous model 2: 2D RadVis.

Since the dimensional anchor G has the highest value of 97, it pulls the node N most strongly, whereas the dimensional anchor D pulls the node N least strongly as it has the lowest value of 7. Similarly, RadVis locates data instances based on the aforementioned principle. When the same principle was applied, the results of 2D RadVis were obtained as shown in Figure 4.

As shown in Figure 4, the nodes representing dementia patients are distributed inside the circle. Based on dimensional anchors located closest to the nodes, we can find that nodes carry higher values on dimensional anchors. In other words, green nodes shown in Figure 4 that represent patient groups in the early phase of dementia are distributed closely to Siadl Sum variables, a daily living test index (the nodes highlighted red on the left side of Figure 4), suggesting that nodes carry a high value in the daily living test index. Thus, we can see whether data instances carry higher or lower values on each variable better than the 2D node-link diagram. However, if the number of nodes increases or the increased number of dimensional anchors leads to additional node-pulling points as shown on the right side of Figure 4, the nodes distributed inside of the RadVis are overlapped or located at the center. Especially, it is difficult to understand pitches of variable node values at the center. We reviewed our study to improve these limitations based on the study of Ibrahim et al. [33,34], which spread the distribution of nodes evenly using Pareto front methods to 3D RadVis model.

#### 3.3.3. Accepted Model: Visualization Combined 3D RadVis and Parallel Coordinates

In this study, we improved the node distribution of data using 3D RadVis technique, which prevented the node overlap during visualization. In addition, it facilitated the distribution of numerous nodes into optimum locations regardless of the number of dimensional anchors. However, in 3D RadVis cases utilizing the preexisting Pareto front methods, each node located in the same space had different values.

In order to correct this phenomenon, the 3D RadVis in this study was designed to locate a node based on average value. Based on this principle, each node never overlaps at the middle and shows an even spread of results through the 3D polygon. Further, utilizing the depth of *z* axis, each node varies in height according to the value of variables. The development of the process and interactions of the final 3D RadVis and Parallel Coordinates will be described in Section 3.4.

### 3.4. Visualization

#### 3.4.1. The Developing Process of Visualization

**Figure 5:**
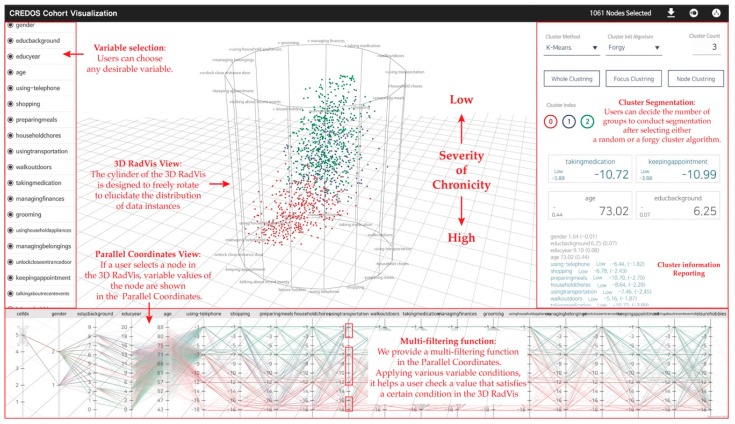
Visualization combining 3D RadVis and Parallel Coordinates.

Following the design process discussed in Section 3.3, this study invented an analytical tool combining the final model based on the interaction between 3D RadVis and Parallel Coordinates. This tool facilitated segmentation of groups by repeatedly clustering data. The visual interface is shown in Figure 5.

In this study, we have improved the layout of 3D RadVis to distribute nodes accurately according to the variables utilizing the rotation of a 3-dimensional polygon and depth of *z* axis. The 3D RadVis expresses the range of values according to the size of each variable represented by the height of *z* axis. The strength of each node inside the polygon is based on the following principle and calculated as follows.

(1) $$N_{p\left(i\right)}=\frac{N_{\left(i\right)}-V_{min\left(i\right)}}{V_{max\left(i\right)}-V_{min\left(i\right)}}\times \left(P_{\max\left(i\right)}-P_{\min\left(i\right)}\right)+P_{min\left(i\right)}\;$$

In the Equation, *N_p(i)_* is the location associated with the ith variable of single node and *N_(i)_* represents the value of ith variable of single node. *V_max(i)_* refers to the maximum value of the ith variables in each node and *V_min(i)_* is the minimum value of the i^th^ variables in each node. Also, *P_max(i)_* denotes the upper bound of the ith variable and *P_min(i)_* is the lower bound of the ith variable. The following effects occur when the node is distributed with this equation.

The strength of each node depends on whether the single variable has a minimum, maximum, or median value. If variable value of node gets near maximum value, node receives strength toward dimensional anchor placed at maximum value. Conversely, if the value of variable reaches the minimum level, the node gains in strength from the midpoint of the polygon bottom. When the value of variable ranges between maximum and minimum, the strength varies linearly and follows the curve (or straight) connecting the minimum and the maximum values. These forces result in the expansion of nodes according to their numeric value while preventing overlap at the central point.

Regarding the dimensional anchor, the variables selected by the user are located among the 62 variables extracted in the CREDOS data. A single node represents a single patient with dementia.

Variables considered as dimensional anchors must be numerical variables (e.g., item variables for the evaluation of dementia patients), while categorical variables such as gender and disease diagnosis are not shown on 3D RadVis. Appropriate comparisons between data according to the height of *Z* axis can only be made using the same scale.

In sequence, we have developed a multifiltering function using the Parallel Coordinates to assign various conditions for comprehensive data analysis. The multifiltering function is designed for applications involving variables with more than two conditions and accordingly, the various values in the data range can be viewed. The Parallel Coordinates can be used to represent both numerical variables and categorical variables.

In the study, we propose a function, which facilitates the segmentation of patients with dementia via repeated data clustering. We applied Random and Forgy algorithms to set the initial focus of the cluster. The random algorithm creates a pivot point inside the 3D RadVis arbitrarily, resulting in cluster variation each time.

However, under similar cluster conditions, the Forgy algorithm always yield identical results, as the specific node selected became the cluster center.

Following clustering with either Random or Forgy algorithm, the central value of the cluster was obtained according to the number of clusters. After the Euclidean distance between central value and each node was calculated, various nodes were included to obtain clusters of similar value. This process was repeated until the central point remained constant.

#### 3.4.2. Visualization Interaction

First, users can choose any desirable variable via “Variable Selection Menu” located in the left side of the analytical tool. Next, the *Z* axis edge of the 3D RadVis and the axis of Parallel Coordinates appear. If users desire to segment clusters of data instances, they can select “Cluster Segmentation Menu” on the right side of the tool. For cluster segmentation, they can decide the number of groups for segmentation after selecting either a random cluster or a forgy cluster algorithm. The segmentations can be repeated until the desirable results are achieved. In the “3D RadVis View”, the cylinder of the 3D RadVis is designed to freely rotate to elucidate the trait distribution of data instances [35].

Further, our tool supports a variety of interactions. To begin with, if a user selects a node inside the 3D RadVis, variable values of the node appear as a line of the Parallel Coordinates. Thus, users can determine the values of a specific node on each variable axis. Moreover, we provide a multifiltering function in the axis of the Parallel Coordinates. Under variable conditions, it allows a user to determine the value that satisfies a specific condition in the 3D RadVis. A user can locate a variable that meets two or more conditions along a single variable axis using a mouse to fix two or more areas on the axis of the Parallel Coordinates dragging.

On the right side of the tool, a section showing index information of the generated clusters provides descriptive statistics of each cluster such as mean and median values. Moreover, users can extract the clustered data into csv extensions for further analysis. The aforementioned interactions were developed according to the proposals of psychiatrists who compared various ranges of each variable.

### 3.5. Qualitative Evaluation (Implimentation of Visualization System)

This study conducted a user evaluation of psychiatrists based on visualization analysis. The test was based on qualitative assessment focusing on questions of subjective opinions using the tool. Subjects participating in the evaluation included medical teams involved in diagnosing and treating dementia patients (one psychiatrist and one clinical psychologist). The psychiatrist has 20 years of research and medical diagnostic experience, and the clinical psychologist has 10 years of research experience. The participants are whom mainly focused on research such as Dementia, elderly mental health and elderly behavior. The evaluation entailed the following steps: implementing the visual analytical tool, conducting assigned tasks, and interviewing. These steps lasted approximately 90 min per person.

Dementia data variables used in the visualization analysis included 15 questions of S-IADL. The test was composed of “using telephone, shopping, preparing meals, household chores, using transportation, walk outdoors, taking medication, managing finances, grooming, using household appliances, managing belongings, unlock close entrance door, keeping appointment, talking about recent events, and leisure hobbies.” The S-IADL data were scored using the 4-point Likert scale, depending on patients’ responses to each question. The patients’ data were represented by nodes in 3D RadVis. The 3D RadVis was visualized as a 15-dimensional polygon based on the usage of 15 questions in S-IADL as target variables.

First, we informed participants about the characteristics of 3D RadVis and Parallel Coordinates visualization and offered time to experience each visualization.

Next, we checked the usability of their visualization with a simple tutorial. The tutorial included node selection within 3D RadVis and polygon rotation, following instructions to segment cluster and filter the desired range in Parallel Coordinates.

In the main experiment, we trained the participants to operate 3D RadVis and Parallel Coordinates visualization by selecting patient information, disease diagnosis and S-IADL-related variables. The distribution of dementia patients in the 3D RadVis was facilitated by visualization of only 15 questions related to S-IADL. The Parallel Coordinates enables the control of variables selected by the participants.

Also, the task of node segmentation in dementia patients distributed in 3D RadVis was designed to encourage the use of Forgy algorithm of k-means to segment nodes into five clusters. Visualization exploration and cluster segmentation lasted 30 min, and the analysis of each cluster lasted 10 min. Subsequently, each participant was interviewed using the questionnaire listed in Table 3. Evaluation questions were related to the design task (Section 3.2) developed in this study. The assigned tasks are indicated below.

Based on the questionnaire presented in Table 3, the participants’ responses were organized as follows. First, both the psychiatrist and clinical psychologist agreed on the efficiency of data exploration. The cognitive ease of the visualization tool facilitated total data distribution by combining different variables of cognitive test results.

Especially, there was an opinion that during the exploration of 3D RadVis, the ability to observe the node while freely rotating the polygon is a great advantage. The psychiatrist participant had experienced 2D RadVis visualization before, but then, when a bunch of variables were selected together, an overlapping of nodes occurred, which eventually prevented readability and node selection. However, by using the newly designed 3D RadVis system, the cluster of nonoverlapping nodes containing similar characteristics was seen. Further, the height of each node inside the 3D polygon facilitates intuitive understanding of the overall dementia progress.

Also, participants compared the nodes in close proximity by comparing the differences in the variable value based on the line graph of Parallel Coordinates. In addition to analyzing patients’ information via nodes presented in the 3D RadVis, they frequently examined the nodes of RadVis after selecting the range of values in the specific tests based on line graphs of the Parallel Coordinates. This finding suggests that the visualization system was user-friendly.

Also, clustering and segmentation based on various variables were considered helpful for medical data analysis. Visual analysisis is an efficient tool for data assessment and segmentation.

The disadvantages include the long learning curve required to use the tool. Since subjects had no prior experience with visual analytical tools, they faced difficulties with data interpretation at first. Moreover, they encountered considerable difficulties understanding the task of “efficiently exploring the closest nodes” despite adequate training before the evaluation process. Therefore, we concluded that there was an entry barrier to users unfamiliar with visualization.

Nevertheless, the two psychiatrists analyzed the data of dementia patients (Table 3). In particular, based on the task used to select patients according to the most common test results in the MCI stage cluster, we confirmed the value representing each cluster correctly because participants selected nodes that were at or near the center of mass.

**Figure 6:**
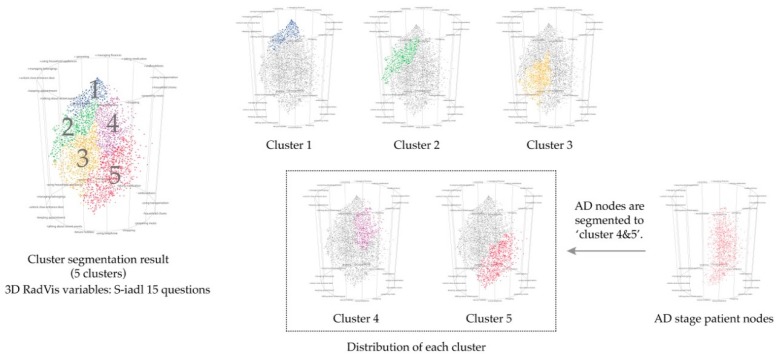
Result of segmenting dementia patient group into 5 clusters.

During cluster comparison and segmentation, as well as MCI stage cluster, the task was resolved by participants who compared the distribution location of the two clusters and the line patterns of the Parallel Coordinates. As a result, the patterns derived from the two clusters were similar, which indicated the results obtained via cluster segmentation similar to the diagnostic features of existing dementia. The results of participant progress through cluster segmentation are shown in Figure 6.

Based on the results of authentication, the segmented group in clusters 4 and 5 (Figure 6) turned out to be clusters belonging to the Alzheimer’s disease (AD) stage, which is one of the diagnostic steps in the evaluation of existing dementia. The clusters belonging to the AD stage showed patients with high and low severity of chronicity, which prevented a definitive diagnosis when treated with medications associated with insignificant effect.

However, using cluster segmentation, a meticulous diagnosis will be able to obtain for each group according to the severity of chronicity even if patients were at a similar AD stage. Also, further segmentation of the AD levels and comparison of their activities of daily living were suggested.

Following qualitative evaluation, participants provided additional feedback on the functions and direction of the tool. First, in order to visualize 15 questions of S-IADL using the tool, it was proposed that each question should be classified in accordance with physical and psychological functions used to conduct the suggested behaviors. Following this suggestion, one of the S-IADL questions involved “cooking”, which was classified as a behavioral item because it depended on memory, behavioral insight, and recognition. Based on this detailed classification, it is necessary to identify the physical and psychological elements that exhibit a close relationship with comparatively lower daily life behaviors. Moreover, the ongoing studies involving detailed segmentation of patients with early dementia in the field of neuropsychology suggest that their classification facilitated research efforts. Second, a meaningful cluster analysis via analysis of dementia patient data is necessary to develop independent clusters of patients, without relying on existing dementia diagnostic indices (for SMI through AD). This feedback was provided because we proposed the task according to the existing index during qualitative evaluation.

## 4. Case Study

This case study was designed to assist medical experts understand the benefits of visualization analysis to segment patients with Alzheimer’s disease showing a comparatively high level of dementia based on S-IADL questionnaire included in the CREDOS. The case study was prompted by psychiatrists who showed an interest in diagnosing Alzheimer’s disease (AD Stage) based on S-IADL.

The case study was conducted because participants undergoing qualitative evaluation (Section 3.5) desired to further classify patients into segmented clusters and evaluate differences in activities of daily living. In this case study, we segmented patients with AD into 3 groups for analysis. At this time, the axis of 3D RadVis was set based on 15 variables in the S-IADL questionnaire. The Parallel Coordinates graph includes 20 variables such as dementia diagnostic steps, gender, academic background, education year, age, and 15 questions related to S-IADL.

**Figure 7:**
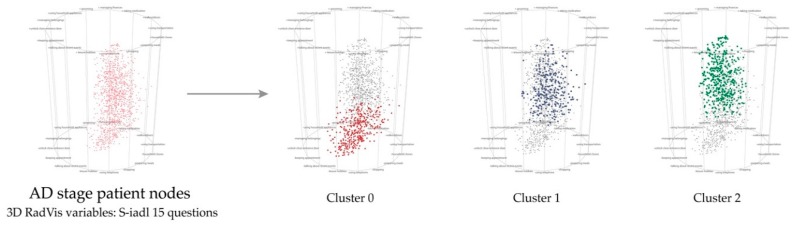
The result of segmenting patients on AD stage into 3 groups.

**Figure 8:**
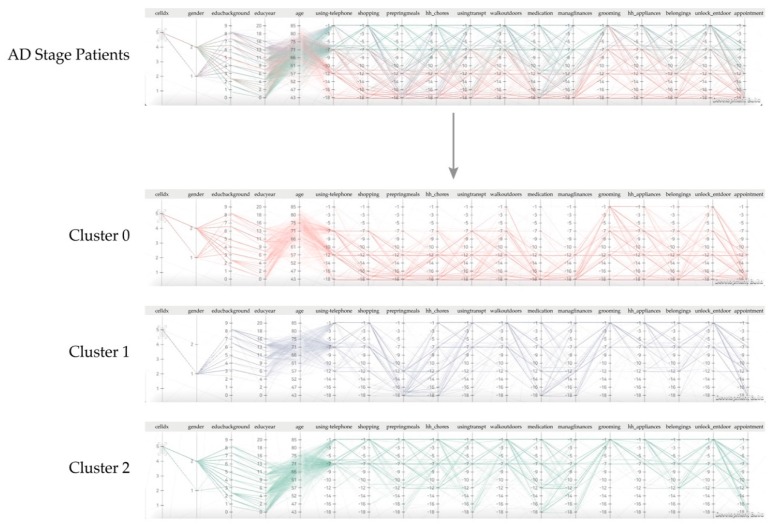
The result of AD stage segmentation using the Parallel Coordinates graph.

Figure 7 presents the distribution of AD patient group segmented with 3D RadVis. Figure 8 presents the characteristics of AD patient group segmented into 3 clusters.

Based on the analysis of results using the two types of visualization, the high-risk group (cluster 0) of AD patients showed abnormal symptoms in response to questions about shopping, using transportation, walking outside, and locking doors. However, in the low-risk group (cluster 2), abnormal symptoms were recorded in response to questions related to treatment with medications, financial management, upholding promises, and discussing recent events. An abnormal symptom was indicated by a low score in Parallel Coordinates, and a score closer to −17 represented a low score. Further, each group of AD patients consistently scored low on individual factors (maintaining appointments, talking about recent events, and leisure hobbies). The results of this case study were discussed with psychiatrists, who agreed that factors related to human relationship may affect the progression of dementia.

When the line graph distribution of each cluster of Parallel Coordinates was analyzed, the clusters 1 and 2 were seen at both extremes, which suggested a clear difference in the degree of distribution of test response values. The variable involving S-IADL showing these differences are question to “shopping, getting ready for meal, doing the chores, using public transport, taking a walk, taking medicine, managing financial, dressing up.” Based on the differences in data distribution, the clusters 1 and 2 can be distinguished, whereas cluster 0 contains the characteristics of both clusters 1 and 2. Therefore, clusters 1 and 2 can be distinguished from other clusters. With regard to the question on ‘grooming’, all the three segmented groups scored differently. The score was mostly −1 and −7 in cluster 0, but −1, −7, −12, and −16 in cluster 1, and −1 in cluster 2. Interpreting each result value, the cluster 0 included many patients who struggled with grooming, cluster 1 contained many patients who often showed difficulty with grooming, and cluster 2 involved many patients who never had any challenges with grooming. Thus, the clusters showed a distinct difference in grooming ability based on their response to 15 question in the S-IADL questionnaire. As a result, our study case showed daily actions that affected the progress of AD, but manifested slight differences in worsening behavior according to the chronicity of the disease.

## 5. Conclusions

The aim of this study was to establish a tool based on visual analysis for domain experts such as psychiatrists to stratify and diagnose patients with dementia. To this end, we combined 3D RadVis and Parallel Coordinates.

The contributions of this study are as follows. First, it proposed a visualization tool for analysis of multidimensional medical data via collaboration with psychiatrists and established its utility for further analysis of medical data. Second, the visualization tool proposed in this study allowed an even distribution of data by adopting a dimensional anchor based on *z* axis edge to prevent node overlapping in 3D RadVis visualization. Because data analysis can be distorted due to node overlapping, the method is expected to facilitate data analysis by psychiatrists.

Particularly, the cluster segmentation function in our system provides insight into the character of dementia patients and demonstrates that patients with AD manifesting a relatively wide range of chronicity can be segmented according to their daily activities. These results contributed to a positive outlook among domain experts, who may be encouraged to recommend sophisticated prescriptions for each of the subdivided groups. This outcome was confirmed via a qualitative evaluation and the case study.

However, the study limitation involved failure to conduct interviews with medical teams from diverse fields (clinical psychologists and psychiatrists). Moreover, as the number of participants in the qualitative evaluation was less than 5, it was hard to generalize the efficacy of the system with collected opinions. Thus, it is necessary to expand the number of subject experts when conducting qualitative evaluation in further studies. The visual tool can be improved by adding and elaborating functions based on participants’ comments.

## Figures and Tables

**Table 1:** System design guideline.

Design Task	Explanation
Understanding the representitivness of clusters	1. Can psychological test values of patients with general symptoms of MCI ^a^ represent the whole MCI groups? 2. What are the differences in daily living between MCI and AD ^b^ groups?
Efficiently exploring the closest nodes	1. How can we find the patients with a daily living test score above N among SMI ^c^ patients?
Segmenting and parting dementia patient groups	1. Is there a score difference between psychologic tests among the segmented groups? If so, which symptoms show the largest difference?

^a^ MCI—Mild Cognitive Impairment; ^b^ AD—Alzheimer’s Disease; ^c^ SMI—Subjective Memory Impairment.

**Table 2:** Data components included in Clinical Research Center for Dementia of South Korea (CREDOS). Reproduced with permission from the authors of [10]; published by ACM, 2017.

Variables	Explanation
Patient information	Cohort ID, personal information (gender, age, educational background), physical examination
Caregiver information	Caregiver’s information (gender, age, educational background, relationship between patient and caregiver)
Cognitive assessments	Caregiver-Administered Neuropsychiatric Inventory
	(CGA-NPI), Seoul-Instrumental Activities of Daily Living (S-IADL), diagnosed disease (SMI ^a^, MCI ^b^, VCI ^c^, SVD ^d^, AD ^e^)

^a^ SMI—Subjective Memory Impairment; ^b^ MCI—Mild Cognitive Impairment; ^c^ VCI—Vascular Cognitive Impairment; ^d^ SVD—Subcortical Vascular Dementia; ^e^ AD—Alzheimer’s Disease.

**Table 3:** Participant questionnaire for qualitative evaluation.

Topic (Based on Design Task)	Questionnaire List
Understanding the representitivness of clusters	1. (Based on k-means cluster (forgy) analysis, who is a typical patient carrying the most general test results in the MCI ^a^ cluster? 2. Assume that you have selected one of the clusters analyzed via k-means (forgy). Based on your empirical experiences, can the selected group represent the traits of MCI patients?
Efficiently exploring the closest nodes	1. Based on the selection of a certain cluster, what can you tell about the nodal traitsdistributed on each pole of a cluster?
Segmenting and parting dementia patient groups	1. Based on your empirical experiences, what do you think of the clusters of dementia patients derived from k-means? 2. Do you think the number of segmented clusters (5) are adequate for the data type?

^a^ MCI—Mild Cognitive Impairment.
